# β-Catenin Signaling Increases during Melanoma Progression and Promotes Tumor Cell Survival and Chemoresistance

**DOI:** 10.1371/journal.pone.0023429

**Published:** 2011-08-17

**Authors:** Tobias Sinnberg, Moritz Menzel, Daniel Ewerth, Birgit Sauer, Michael Schwarz, Martin Schaller, Claus Garbe, Birgit Schittek

**Affiliations:** 1 Division of Dermatooncology, Department of Dermatology, Eberhard-Karls-University Tübingen, Tübingen, Germany; 2 Department of Toxicology, Institute of Pharmacology and Toxicology, University of Tübingen, Germany; 3 Department of Dermatology, Eberhard-Karls-University Tübingen, Tübingen, Germany; Northwestern University Feinberg School of Medicine, United States of America

## Abstract

Beta-catenin plays an important role in embryogenesis and carcinogenesis by controlling either cadherin-mediated cell adhesion or transcriptional activation of target gene expression. In many types of cancers nuclear translocation of beta-catenin has been observed. Our data indicate that during melanoma progression an increased dependency on the transcriptional function of beta-catenin takes place. Blockade of beta-catenin in metastatic melanoma cell lines efficiently induces apoptosis, inhibits proliferation, migration and invasion in monolayer and 3-dimensional skin reconstructs and decreases chemoresistance. In addition, subcutaneous melanoma growth in SCID mice was almost completely inhibited by an inducible beta-catenin knockdown. In contrast, the survival of benign melanocytes and primary melanoma cell lines was less affected by beta-catenin depletion. However, enhanced expression of beta-catenin in primary melanoma cell lines increased invasive capacity in vitro and tumor growth in the SCID mouse model. These data suggest that beta-catenin is an essential survival factor for metastatic melanoma cells, whereas it is dispensable for the survival of benign melanocytes and primary, non-invasive melanoma cells. Furthermore, beta-catenin increases tumorigenicity of primary melanoma cell lines. The differential requirements for beta-catenin signaling in aggressive melanoma versus benign melanocytic cells make beta-catenin a possible new target in melanoma therapy.

## Introduction

The canonical Wnt/β-catenin signaling pathway plays a key role in embryogenesis and cellular homeostasis and regulates cell fate, differentiation, proliferation and self-renewal of stem cells and progenitor cells. The activity of the central signaling molecule β-catenin is mainly determined by regulation of proteolysis by the β-catenin destruction complex including the principal constituents casein kinase 1α (CK1α), glycogen synthase kinase 3 (GSK3), adenomatous polyposis coli (APC) and axin [1], [2]. In the absence of Wnt pathway activation, cytosolic β-catenin is phosphorylated and targeted for degradation. On Wnt stimulation the β-catenin destruction complex dissociates, leading to an accumulation and nuclear translocation of β-catenin, which is followed by binding to the T cell factor/lymphocyte enhancer binding factor family (TCF/LEF) and transcription of β-catenin/TCF/LEF responsive genes [3]. Stabilization or nuclear translocation of β-catenin has been observed in many types of cancers, such as colon, lung, skin, breast, liver and pancreas cancers. Especially, truncating mutations of the tumor suppressor APC are the most prevalent genetic alterations in colorectal carcinomas [4].

Several studies have shown that the Wnt/β-catenin signaling pathway regulates formation of neural-crest derived melanocytes and by this influences melanocyte development [5], [6]. Furthermore, in a transgenic mouse model it was shown that β-catenin promotes immortalization of murine melanocytes by suppression of the tumor suppressor p16INK4A and cooperates with N-Ras in melanoma development [7], [8]. In malignant melanoma, however, there are contradictory results concerning the role of β-catenin in tumor progression. Whereas several studies show nuclear accumulation of β-catenin in at least 30% of melanoma cells [9]–[13] and propose that an increased nuclear translocation and activity of β-catenin promote melanoma proliferation [14], [15], others found that elevated levels of nuclear β-catenin correlate with improved survival of melanoma patients [16] and that β-catenin downregulation promotes metastases formation in mice [17]. From these data it has been proposed that canonical Wnt signaling via activation of β-catenin is required for melanoma genesis, whereas its continued expression in later stages inhibit metastases formation. On the other hand, non-canonical Wnt signaling, specifically Wnt5A, influences canonical pathways by downregulation of β-catenin and signals to promote melanoma metastasis [16]–[20].

We recently described that melanoma cells developed an efficient new mechanism to activate the β-catenin signaling pathway by suppression of CK1α expression, defining CK1α as a novel tumor suppressor in melanoma [21]. We observed that in benign melanocytic cells and primary melanoma cells expressing high levels of CK1α, β-catenin is mainly localized at the cell membrane and that the free cytoplasmic and nuclear pools of β-catenin increase during melanoma progression, in particular due to downregulation or loss of CK1α expression. These studies suggested that there is a differential dependency of benign melanocytic cells and primary melanoma cells on β-catenin concerning proliferation and survival. Based on these results we asked in this study whether β-catenin fulfills different roles in benign melanocytic cells and non-invasive primary melanoma and metastatic melanoma cell lines.

## Results

### β-catenin is an essential survival factor for metastatic, but not for primary melanoma cell lines and benign melanocytes

**Figure 1:**
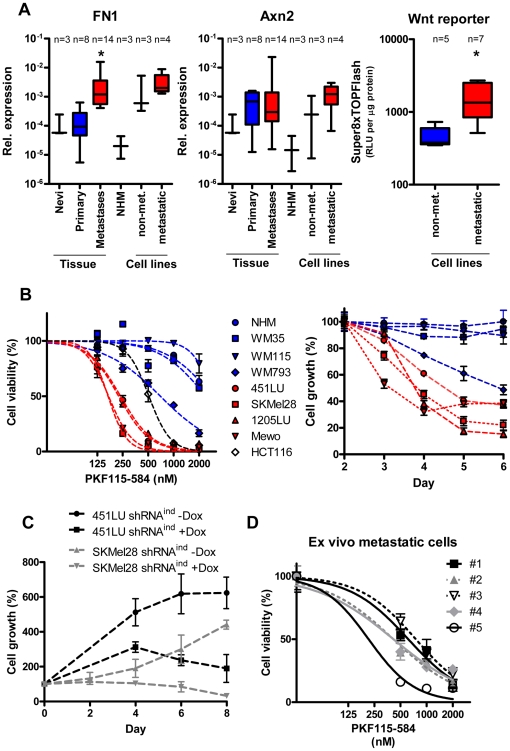
β-catenin is a survival factor for metastatic, but not for primary melanoma cells and benign melanocytes. (**A**) *β-catenin signaling activity in melanoma samples:* mRNA expression of the Wnt target genes fibronectin (FN1) and axin2 (Axn2) was measured in melanoma tissue samples and benign nevi as well as in non-metastatic, or metastatic melanoma cell lines and normal human melanocytes (NHM) by real-time PCR (normalized to β-actin). The canonical Wnt signaling activity in melanoma cell lines was assayed with the Super8xTOPFlash reporter plasmid. Luciferase signals (RLU) were normalized to protein amount and measured in metastatic and non-metastatic melanoma cell lines (right diagram). Blue bars: benign melanocytes, nevi and primary, non-metastatic melanomas; Red bars: metastatic melanomas. In melanoma metastases FN1 was found to be significantly overexpressed compared to non-metastatic samples (p = 0.045). Wnt reporter activity was significantly higher in metastatic compared to non-metastatic melanoma cell lines (p = 0.042). Significant differences are marked with asterisk (student t-test (95% CI)). (**B**) *Influence of β-catenin inhibition on viability/cell growth:* Left panel: Normal human melanocytes (NHM), the primary melanoma cell lines WM35, WM115 (radial, non-invasive growth phase) and WM793 (vertical, invasive growth phase) as well as the metastatic melanoma cell lines 451LU, SkMel28, 1205LU and Mewo were assayed in a fluorescence cell viability test after three days of treatment with increasing concentrations of the small molecule PKF115–584. The colon cancer cell line HCT116 served as a control. Curve fits were calculated using a sigmoid dose response model with variable Hill slope. Right panel: Shown is the time-dependent cell number (in % of control) after shRNA mediated downregulation of β-catenin. Lentiviral shRNA against β-catenin was used to transduce the indicated cell lines. Due to the DsRed cassette in the lentiviral transfer plasmid, the fluorescence of transduced cells was used for proliferation measurement starting 2 days after transduction. The fluorescence signal was normalized to the fluorescence signal of sh*luc* control transduced cells for every time point. NHMs and non-metastatic melanoma cell lines are marked in blue, non-metastatic melanoma cell lines in red. (**C**) *Doxycyline-inducible shRNA against β-catenin:* Two clones, stably expressing a doxycycline-inducible shRNA against β-catenin were generated from the parental cell lines SKMel28 and 451LU, induced with 1 µg/ml doxycycline and counted every 48 hours in triplicates to monitor cell proliferation. The relative cell growth of the doxycycline induced samples compared to the non-induced populations are shown (normalized to the number of seeded cells). (**D**) *Pharmacological β-catenin inhibition in patient melanoma skin metastases:* Five isolated melanoma cell populations from melanoma skin metastases were tested in a fluorescence cell viability assay (MUH) after three days of treatment with increasing concentrations of PKF115–584.

We observed that RNA expression of the β-catenin target genes fibronectin (FN1) and axin (Axn2) were upregulated in primary melanomas and melanoma metastases compared to benign nevi (**Figure 1A**). In a similar way metastatic melanoma cell lines showed a higher RNA expression of both genes compared to non-metastatic melanoma cell lines and normal human melanocytes (NHM). Furthermore, β-catenin-TCF/LEF signaling activity was higher in metastatic compared to non-metatastatic melanoma cell lines as determined by a Super8xTOPflash reporter assay (**Figure 1A**). These data suggest that the transcriptional activity of β-catenin increases during melanoma progression.

To evaluate the role of β-catenin during melanoma progression we downregulated β-catenin in several well-defined human melanoma cell lines derived either from primary melanomas or from melanoma metastases [22]–[24] using either a small-molecule inhibitor of β-catenin/TCF/LEF complexes (PKF 115–584) [25] or shRNA against β-catenin (**Figure 1B**). PKF115–584 treatment of several melanoma cell lines and the colon cancer cell line HCT116 as a control was not only able to downregulate β-catenin transcriptional activity, but also β-catenin expression in a dose-dependent manner (**Figure S1A and S1B**). Furthermore, expression of either lentivirally delivered shRNA against β-catenin or doxycycline-inducible knockdown of β-catenin using shRNA efficiently reduced the amount of β-catenin protein (**Figure S2A and S2B**). Inhibition of β-catenin expression and signaling in metastatic melanoma cell lines (451LU, 1205LU, Mewo, SKMel28) using PKF115–584 treatment resulted in a dose-dependent reduction of viability (**Figure 1B**) accompanied with morphological changes, such as rounding up and detachment from the culture plate (data not shown). In contrast, the viability of normal human melanocytes (NHMs) and the primary melanoma cell lines WM35 and WM115 was completely unperturbed by the inhibition of β-catenin (**Figure 1B**). To analyze whether proliferation is affected we downregulated β-catenin using shRNA. Here, a significant growth inhibition of metastatic melanoma cell lines was measured up to 8 days post transduction with the lentiviral shRNA, whereas the non-metastatic cell lines behaved similiar to the non-silencing shRNA control transduced cells (**Figure 1B** **, Figure S4**). Furthermore, knock down of β-catenin expression in metastatic melanoma cell lines using doxycycline-inducible shRNA significantly inhibited proliferation of the metastatic melanoma cells lines SKMEL28 and 451LU (**Figure 1C**). Finally, PKF115–584 treatment of melanoma cells, freshly isolated and established from five different cutaneous melanoma metastases, resulted in a concentration-dependent inhibition of cell viability comparable to the effects observed in the metastatic melanoma cell lines (**Figure 1D**). The PKF115–584 concentrations which inhibit 50% of cell viability (IC_50_) are summarized in **Table 1**. These data point out that β-catenin is an essential survival factor for metastatic, but not for primary melanoma cells and benign melanocytes.

**Table 1:** IC_50_ values (in nM) of PKF115–584 treated melanoma cell lines or primary cells (skin metastases or benign cells) calculated using a non-linear regression analysis with variable slope.

Cell lines						
*non-metastatic*			*metastatic*			
WM35	WM115	WM793	SKMel28	Mewo	1205LU	451LU
>2000	>2000	745	168	170	235	224

Primary cell cultures							
*Skin metastases*					*Benign cells*		
#1	#2	#3	#4	#5	NHM	Keratinocytes	Fibroblasts
614	414	751	415	214	>2000	>2000	>2000

### β-catenin downregulation inhibits invasive tumor growth of primary and metastatic melanoma cell lines

**Figure 2:**
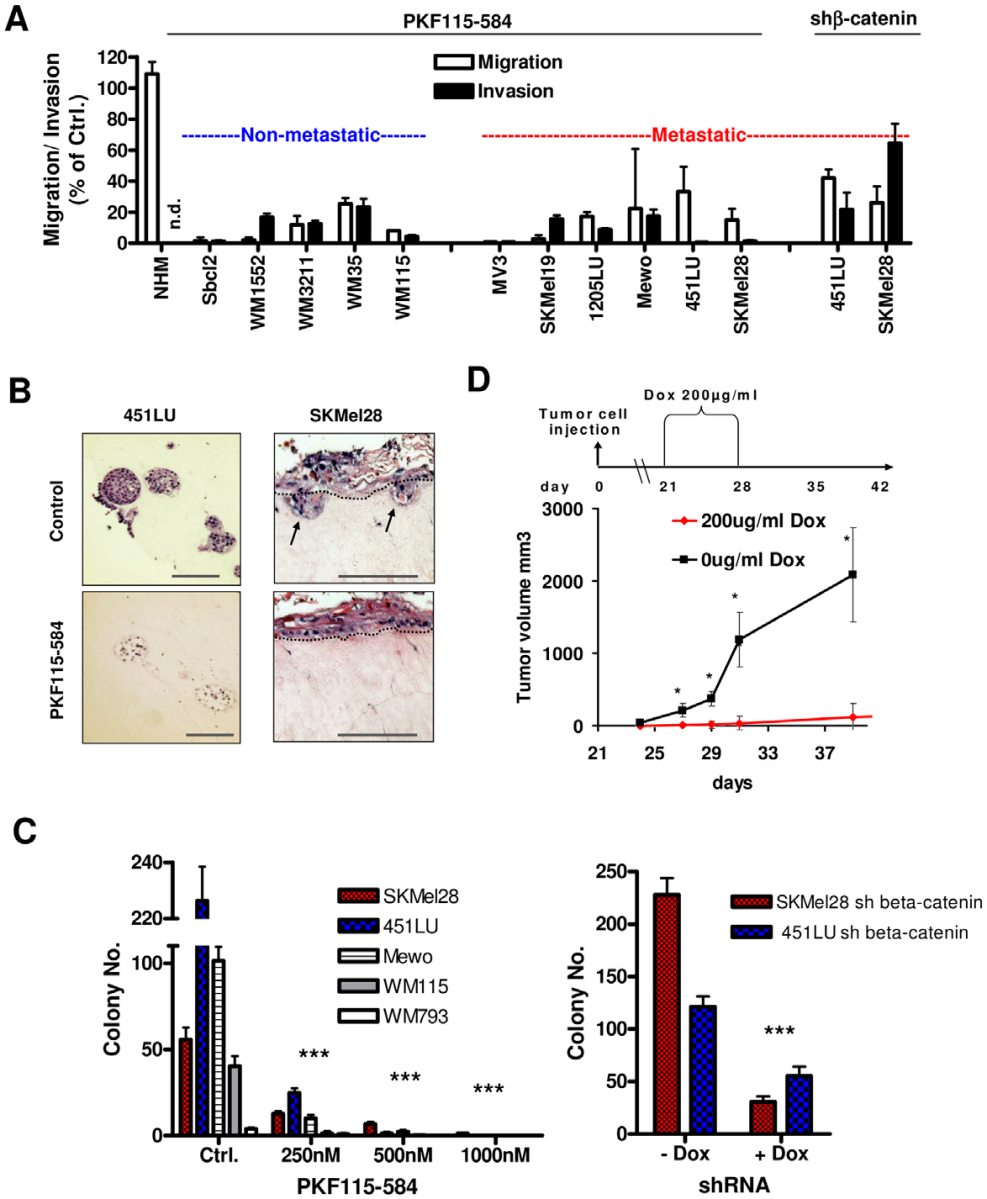
Ablation of β-catenin activity in melanoma cells inhibits migration, invasion, the tumorigenic potential and melanoma formation in vivo. (**A**) *Migration and Invasion:* NHMs and primary melanoma cell lines (left panel, blue bars) or metastatic melanoma cell lines (middle and right panel, red bars) were used to perform Boyden-chamber experiments. Matrigel coated transwells were used to perform invasion assays and non-coated transwells to assay the cell migration capability after treatment with 0.5 µM PKF115–584 for 12 hours (left and middle panel) or the induced expression of shRNA against β-catenin for 24 hours (right panel), a time point at which there is no effect on cell viability as determined by trypan blue staining. Shown is the percentage of migration or invasion compared to the control-treated cells. In each case the same number of viable cells was applied to the chambers. Experiments were done in triplicates. All reductions in migration and invasion were significant except for NHM (p<0.05). ND: not detectable. (**B**) *Organotypic skin reconstruct:* Organotypic dermal (451LU) or epidermal (SKMel28) skin reconstructs were prepared from the metastatic 451LU or SKMel28 melanoma cells. Skin reconstructs were fixed, paraffine embedded and thin sections were stained with hematoxylin/eosin to visualize melanoma cell nests. Arrows indicate invaded melanoma cells. Left panel: Dermal skin reconstruct with 451LU cells. Skin reconstructs were treated with 0.5 µM PKF115–584 for seven days after an initial reconstruct cultivation time of 5 days. Right panel: Epidermal skin reconstruct with SKMel28 cells expressing the doxycycline-inducible shRNA against β-catenin. Epidermal reconstrucs were treated for 10 days with 1 µg/ml doxycycline for shRNA induction. (**C**) *Soft Agar Growth:* Adhesion independent cell growth of the metastatic melanoma cell lines SKMel28, 451LU or Mewo and non-metastatic cell lines WM115 and WM793 (left diagram) or the shbeta-catenin inducible cell clones (right diagram) was measured in a 0.5% agar gel after 14 days of culture with increasing concentrations of PKF115–584 in the feeding layer. Colony numbers were counted after 14 days. The assay was done in triplicates. (**D**) *In vivo melanoma cell growth:* 451LU melanoma cells expressing the doxycycline-inducible shRNA against β-catenin were injected into the flanks of 12 SCID mice which induced small tumor nodules after 3 weeks post injection. One group received 200 µg/ml doxycycline into their drinking water for 7 days (from day 21 to day 28), whereas the control group received 0 µl/ml doxycycline. Tumor growth was measured in total for 40 days post injection of the cells. Asteriks denote significant differences as measured by t-test with one asteriks being p<0.05 and three asteriks being p<0.005 (t-test).

Next, we investigated whether β-catenin has an influence on the migratory and invasive as well as the tumorigenic potential of primary and metastatic melanoma cell lines. Downregulation of β-catenin in six different metastatic and five primary melanoma cell lines using PKF115–584 treatment resulted in a nearly complete abrogation of migration and invasion using a Boyden chamber assay with or without Matrigel as a substrate, whereas migration of NHMs was not significantly affected after PKF115–584 treatment (**Figure 2A** **, Figure S5**). Similarly, the inducible shRNA against β-catenin strongly reduced melanoma cell migration and showed a reduced invasiveness after shRNA induction compared to the non-induced cells (**Figure 2A**). In addition, downregulation of β -catenin by PKF115–584 treatment or by a doxycycline-inducible shRNA against β-catenin inhibited metastatic melanoma cell growth in a dermal environment and prevented invasive growth through the basement membrane at the epidermal dermal junction using an epidermal organotypic skin reconstruct (**Figure 2B**). Furthermore, the tumorigenic potential of non-metastatic (WM115, WM793) and metastatic (SKMel28, 451LU, Mewo) melanoma cell lines in a soft agar growth assay was efficiently inhibited in a PKF115–584 dose-dependent manner (**Figure 2C**). We confirmed this by inducible expression of shRNA against β-catenin in SKMel28 and 451LU cells (**Figure 2C**). These data show that β-catenin downregulation in primary and metastatic melanoma cell lines inhibit migration and invasion in monolayer culture as well as in physiological skin equivalents. Furthermore, it inhibits the tumorigenic potential of non-metastatic and metastatic melanoma cell lines.

### β-catenin expression influences melanoma cell growth in vivo

To analyze the effect of β-catenin downregulation on growth of melanoma cell lines *in vivo*, we subcutaneously injected 2×10^5^ 451LU melanoma cells, in which β-catenin expression can be inducibly knocked down by doxycycline treatment, into SCID mice. 21 days later one group of mice received doxycycline in the drinking water for 8 days, whereas the other group got normal drinking water. Tumor volume was measured starting on day 24 for 14 days. As shown in **Figure 2D** downregulation of β-catenin resulted in a nearly complete inhibition of tumor growth *in vivo*. These data show that β-catenin downregulation in a metastatic melanoma cell line inhibits melanoma cell growth *in vivo*.

**Figure 3:**
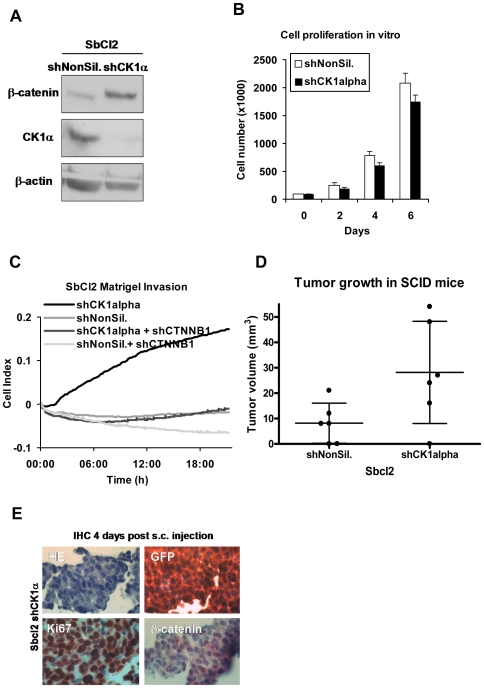
β-catenin expression determines melanoma cell growth in vivo. (**A**) Western Blot analysis of the non-metastatic melanoma cell line SbCl2 after downregulation of CK1α by shRNA (or a non-silencing shRNA as control) with a lentiviral vector and subsequent selection of stable clones. (**B**) The influence on proliferation was assayed by counting the cells every two days revealing no difference in cell growth *in vitro*. (**C**) Stable SbCl2 transductants were seeded onto a CIM plate (XCelligence DP; Roche), coated with Matrigel (1∶10) for measuring invasion. The knockdown induced a β-catenin dependent invasive cell growth through a matrigel matrix in the XCelligence DP system as indicated by the increase of the cell index (upper right diagram). (**D**) Subcutanous injection of SbCl2 control cells or those in which β-catenin expression was upregulated by shRNA against CK1α into SCID mice revealed a higher tumorigenicity and increased the tumor size by three fold (p = 0.043). Shown is the mean tumor size (+/− SD) 4 weeks after injection of 2×10^6^ cells compared to the cells expressing a control shRNA (lower left diagram). Each dot indicates one tumor out of six mice. (**E**) Immunohistochemistry of skin biopsies of mice at day 4 post injection with SbCl2 shCK1α cells showing viable, proliferating (Ki67) tumor cells (GFP positive) with accumulated β-catenin (lower right picture).

To evaluate the functional effects of upregulated β-catenin expression in primary melanoma cells we downregulated CK1α expression in the non-metastatic, radial-growth phase melanoma cell line SbCl2. As previously described by us [21] this results in a stable upregulation of β-catenin protein level due to inhibition of β-catenin degradation (**Fig. 3A, E**). This could not be achieved after overexpression of wildtype β-catenin since it was rapidly degraded (data not shown). Interestingly, whereas cell proliferation *in vitro* was not affected by enhanced β-catenin expression in SbCl2 cells (**Fig. 3B**), matrigel invasion was significantly increased (**Fig. 3C**). This effect could be reversed by simultaneous downregulation of β-catenin using shRNA (**Fig. 3C**). More importantly, tumor growth of SbCl2 cells subcutaneously injected into SCID mice was significantly increased after inhibition of β-catenin degradation (**Fig. 3D**). These data show that β-catenin increases invasive capacity of primary melanoma cells *in vitro* and tumor growth *in vivo*.

### β-catenin downregulation induces apoptosis in metastatic melanoma cell lines

**Figure 4:**
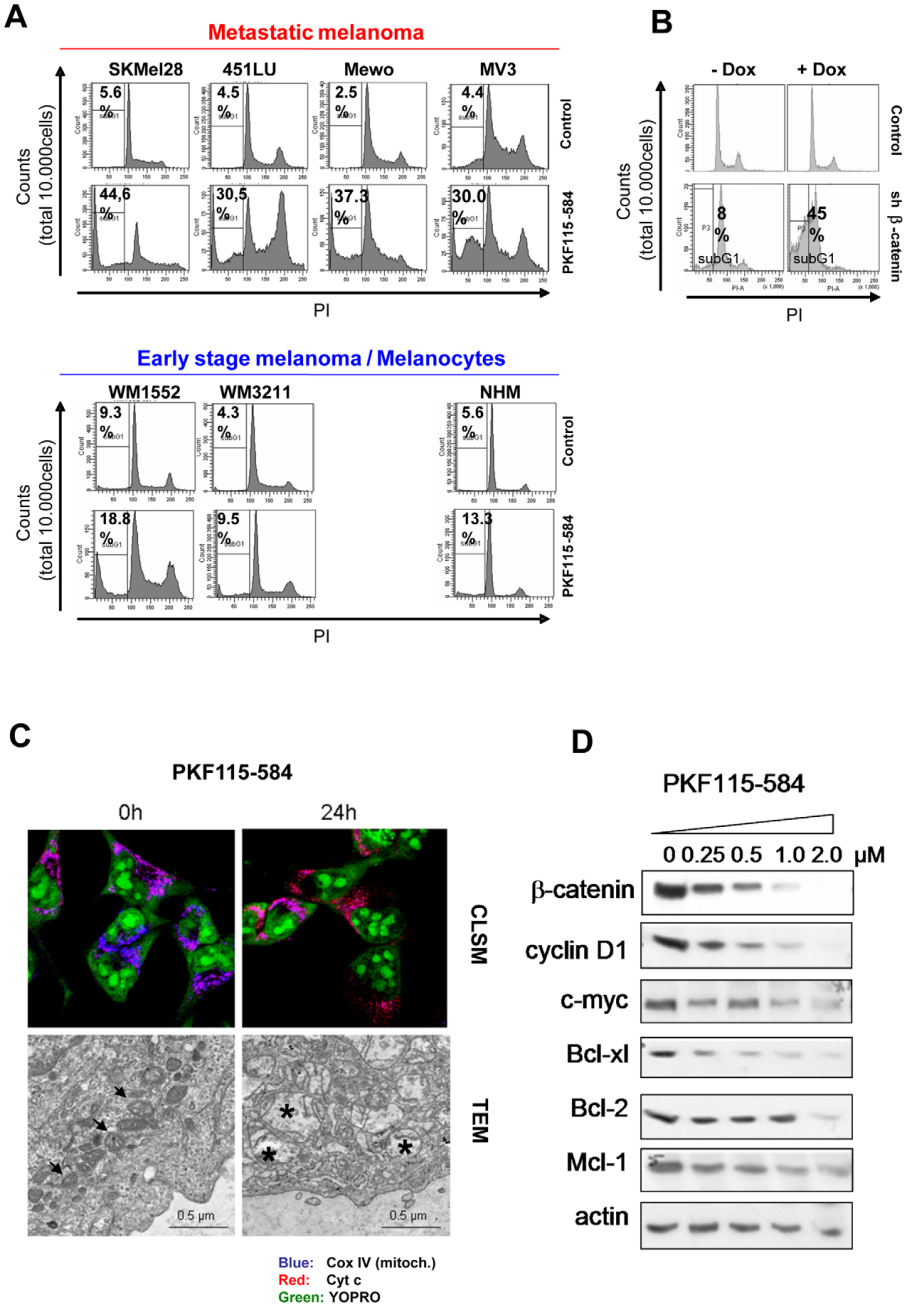
Inhibition of β-catenin induces apoptosis in metastatic melanoma cells. (**A**) Flow cytometric cell cycle analysis by propidium iodide staining of the metastatic melanoma cell lines SKMel28, 451LU, Mewo, MV3, the primary melanoma cell lines WM1552 and WM3211 and NHM 48 hours after PKF115–584 (1 µM) treatment. The percentage of the apoptotic subG1 fraction is indicated. (**B**) Cell cycle analysis of SKMEL28 melanoma cells 8 days after doxycycline-induced expression of shRNA against β-catenin. (**C**) SKMel28 cells were treated with PKF115–584 (1 µM) for 24 hours and either stained for the mitochondrial marker cytochrome c oxidase IV (blue) and cytochrome c (red) plus nuclear staining with YOPRO (green) and analyzed by confocal laser scanning microscopy (CLSM) (top panel) or subjected to transmission electron microscopy (TEM) (lower panel). In untreated cells COXIV co-localizes with cytochrome c, whereas after treatment the COXIV is rarely detectable revealing either damaged mitochondria or cytochrome c release. The electron microscopy revealed massive swollen (>2 fold) mitochondria which occurred already after 12 hours (data not shown). The mitochondria showed degraded cristae and were only weekly electron-dense indicating strong mitochondrial damage. Asteriks/arrows indicate intact/damaged mitochondria. (**D**) Western blot analysis of SKMel28 cells treated with increasing concentrations of PKF115–584 for 24 h for the known β-catenin target genes Cyclin D1 and c-myc as well as the anti-apoptotic Bcl-family members Bcl-2, Bcl-xl and Mcl-1.

To reveal the mechanism of cell death induced by β-catenin downregulation in metastatic melanoma cell lines we analyzed whether apoptosis is induced by β-catenin downregulation using shRNAs or PKF115–584 treatment. Cell cycle analysis showed that β-catenin downregulation leads to a reduced G2/M cell population and the emergence of a subG1 fraction indicative for apoptosis induction (**Figure 4A, B**). We confirmed apoptosis induction by annexinV/PI staining and caspase 3 cleavage (**Figure S3A and S3B**). Furthermore, inhibition of β-catenin induced mitochondrial swelling and the mitochondria showed degraded cristae indicating strong mitochondrial damage and cytochrome C release (**Figure 4C**). In addition, PKF115–584 treatment reduced the expression of the anti-apoptotic proteins Bcl-2, Mcl-1, Bcl-xL and the cell cycle regulator CyclinD1 (**Figure 4D** **, Figure S1B**).

### β-catenin downregulation sensitizes melanoma cells towards chemotherapy

**Figure 5:**
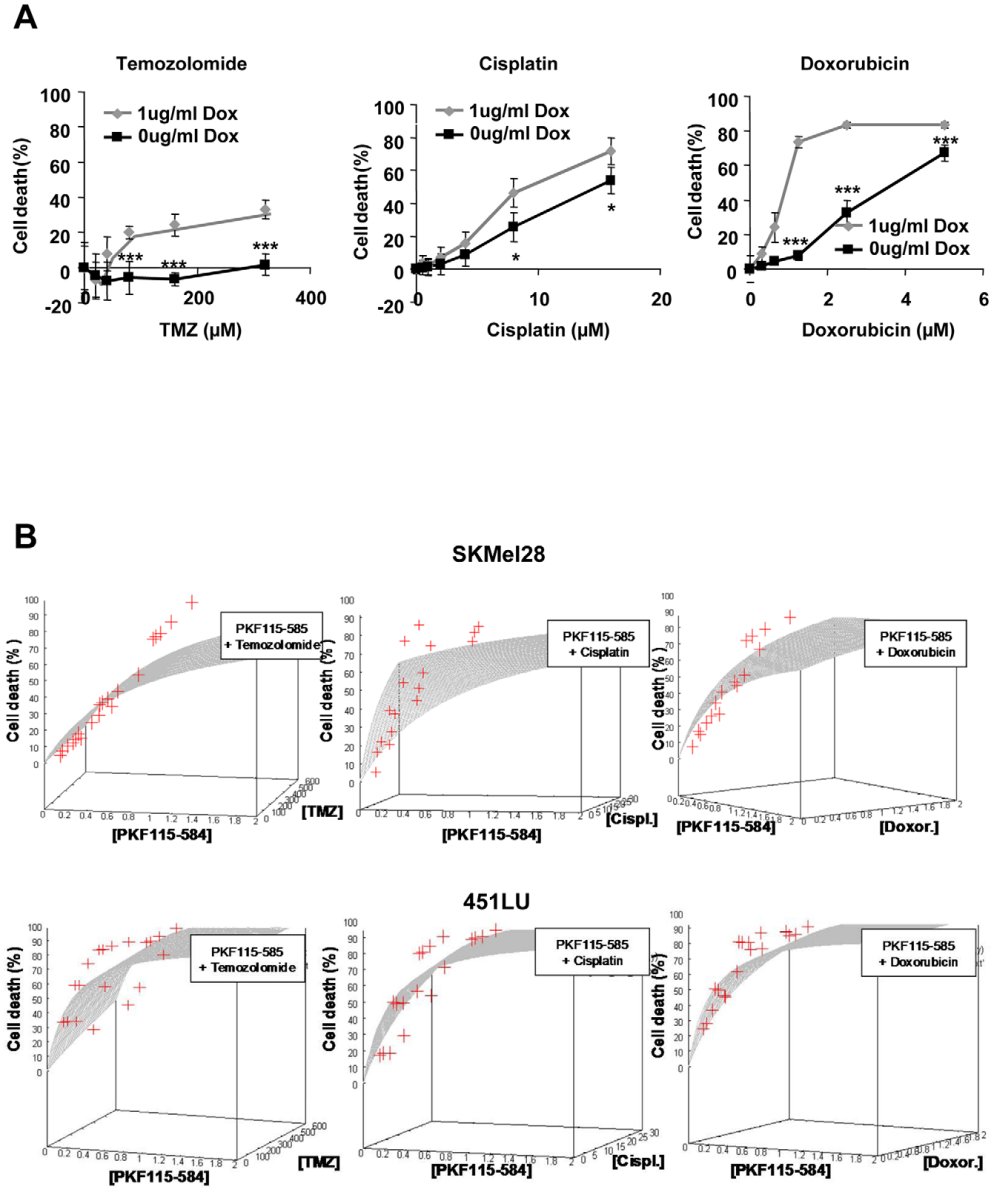
β-catenin increases chemoresistance of melanoma cells. (**A**) 451LU melanoma cells expressing the doxycycline-inducible shRNA against β-catenin were treated with 1 µg/ml doxycycline and 24 hours later with increasing concentrations of the chemotherapeutics temozolomide, cisplatin or doxorubicin for 72 hours. Cell viability was measured 96 hours after shRNA induction. Cell death was calculated out of the viability data and compared to the control. (**B**) 451LU and SKMel28 melanoma cells were treated with increasing concentrations of PKF115–584 (0–1 µM) and the three different chemotherapeutics temozolomide (0–600 µM), cisplatin (0–24 µM) and doxorubicin (0–2 µM) for 72 hours. Cell viability was assessed and the mean values of three independent experiments were used for a response surface analysis to detect synergistic, additive or antagonistic drug combinations. The measured effects were plotted together with the theoretical additive surface in a three dimensional coordination system (x: PKF115–584; y: chemotherapeutic; z: cell death). At concentrations of PKF115–584 reducing β-catenin protein level, the combinations acted synergistically. Asteriks denote significant differences as measured by t-test with one asteriks being p<0.05 and three asteriks being p<0.005 (t-test).

Finally, we analyzed the effect of β-catenin downregulation in metastatic melanoma cell lines on sensitivity towards the chemotherapeutic agents temozolomide, cisplatin and doxorubicin (**Figure 5**). Downregulation of β-catenin in 451LU and SKMel28 melanoma cells by shRNA (**Figure 5A**) or PKF-115–584 treatment (**Figure 5B**) significantly increased chemosensitivity towards all three chemotherapeutics. Response surface analysis [26] shows that the β -catenin inhibitor PKF115–584 acts synergistically with the anti-cancer drugs. The effects after PKF115–584 treatment are positioned above the surface of additivity, i.e. the effects of combination treatment are superadditive or synergistic (**Figure 5B**). These data indicate that β-catenin is critically involved in chemoresistance of melanoma cells.

## Discussion

In this study we show that β-catenin is an essential survival factor for aggressive metastatic melanoma cell lines, but is dispensable for proliferation of normal human melanocytes and primary melanoma cell lines. Furthermore, we show that β-catenin is a key factor determining invasive capacity and tumorigenicity of primary and metastatic melanoma cell lines. Finally, our data indicate that downregulation of β-catenin induces apoptosis in metastatic melanoma cell lines and increases chemosensitivity.

Several studies demonstrated that β-catenin activation is a key step in the initial transformation of melanocytes to melanoma. In a transgenic mouse model it was shown that β-catenin promotes an escape of murine melanocytes from senescence by suppression of the tumor suppressor p16INK4A resulting in melanocyte immortalization. Interestingly, β-catenin cooperates with N-Ras to promote melanoma development in mice [7]. Furthermore, it was shown that cutaneous cancer stem cell maintenance is dependent on β-catenin signalling [6], [27]. Ablation of β-catenin expression eliminated these stem cells and resulted in complete regression of squamous cell carcinomas. Our data suggest that β-catenin is a survival factor for metastatic, but not for primary melanoma cell lines and benign melanocytes. This has not been described so far, but there are reports demonstrating the necessity of β-catenin signalling for the survival of epithelial mammary gland cells [28] as well as in multiple myeloma [29]. Additionally it is known that β-catenin regulates the expression of anti-apoptotic genes, e.g. survivin [30] as well as anti-apoptotic members of the Bcl- family [31]. We have found that depletion of β-catenin results in reduced expression of the anti-apoptotic proteins Mcl-1 and Bcl-xl showing the relevance of β-catenin for cell survival of metastatic melanoma cells. On the other hand β-catenin was recently found to have an additional function in microtubuli and centrosome organization during mitosis [32]–[34].

We provide evidence that during melanoma progression the transcriptional activity of β-catenin increases. It has been shown that β-catenin plays a role both in the β-catenin/E-cadherin cell adhesion complex and as a transcriptional regulator in the nucleus upon canonical Wnt signalling [35]. It is well known that during melanoma progression E-cadherin expression is lost [36] and as a consequence of the disrupted high-affinity cadherin/β-catenin interaction β-catenin is released to the cytoplasm and nucleus to increase β-catenin transcriptional activity [37], [38] This point out that as soon as the cell adhesive pressure is lost, i.e. by loss of E-cadherin expression, β-catenin is freed for its function in the cytoplasm and nucleus and this pool is increased. This could ultimately lead to loss of cell-cell-adhesion control, uncontrolled growth and the capability to invade and metastasize.

In invasive breast cancer downregulation of membrane-associated β-catenin results in decreased cell-cell adhesion and increased motility resulting in a higher probability for metastatic disease [39]. In melanoma there is evidence that activated β-catenin is frequently found in migratory phenotypic melanoma cells [40] and that β-catenin activation is important during transendothelial migration of melanoma cell lines [41]. Indeed, we find that β-catenin is essential for the invasive growth of primary and metastatic melanoma cell lines.

PKF 115–584 has been originally described as a small-molecule inhibitor of β-catenin/TCF/LEF complexes blocking the transcriptional activity of β-catenin in the colon cancer cell line HCT116, while β-catenin expression is not affected [25]. However, it was also shown that PKF115–584 treatment of HCT116 disrupted not only the TCF/β-catenin complex, but also interfered with APC/β-catenin complexes suggesting that expression of β-catenin might also be affected. Indeed, we describe that treatment of several melanoma cell lines and HCT116 inhibit both, β-catenin expression and transcriptional activity in a dose-dependent fashion. More recent publications described also that PKF115–584 treatment resulted in a dose-dependent reduction of total beta-catenin protein levels in some acute myeloid leukemia cell lines [42], smooth muscle cells [43] and mouse embryonic fibroblasts [44]. All of the available data indicate that PKF115–584 may be not a selective inhibitor of the TCF/beta-catenin complex, but can inhibit also total beta-catenin protein levels.

It was proposed that an increased nuclear translocation and activity of β-catenin promotes melanoma proliferation [14], [15] and could worsen the survival of melanoma patients [13]. On the other hand, others found that elevated levels of nuclear β-catenin correlate with improved survival of melanoma patients [16] and that β-catenin downregulation promotes metastases formation in a mouse model [17]. In our studies we find that the free cytoplasmic and nuclear pools of β-catenin increase during melanoma progression, which is then followed by a switch in β-catenin-mediated signaling [21]. We suggest that early in melanoma progression benign nevus cells and primary melanoma cells have a β-catenin pool localized mainly at the cell adhesion complex resulting in increased intercellular adhesion and limited invasive growth potential. During melanoma progression the pool of cytoplasmic and nuclear β-catenin protein levels increase mainly due to loss of CK1α activity which results in a higher β-catenin transcriptional activity [21]. Microphthalmia-associated transcription factor (MITF) as one important transcriptional target of β-catenin in melanoma cells could be critically involved in survival and proliferation of metastatic melanoma cells with high β-catenin transcriptional activity [15], [45].

Altogether we propose that there is a differential dependency of benign melanocytic cells and primary melanoma and metastatic melanoma cell lines on β-catenin concerning proliferation and survival. Targeting of β-catenin inhibits proliferation in all of the metastatic melanoma cell lines tested and migration and invasion of all melanoma cell lines tested (primary and metastatic), whereas proliferation and migration of benign melanocytes are not affected. Several other studies showed that the loss of β-catenin in a number of differentiated cell types is compatible with normal cell viability and tissue structure. Posthaus et al. showed that β-catenin signaling is dispensable during proliferation and early terminal differentiation of epidermal keratinocytes [46]. In teratocarcinoma cells it was shown that the negative effect on cell-cell adhesion by a loss of β-catenin is compensated by an increased expression of plakoglobin [47]. First approaches for the usage of the β-catenin inhibitor PKF115–584 in mouse models showed no severe side effects [48]–[50] pointing out that targeting of β-catenin is feasible and not toxic for the organism. We propose a mechanism by which β-catenin's transcriptional function gets activated during melanoma progression and acts as a survival signal in metastatic melanoma cells. Ablation of β-catenin's transcriptional activity induces downregulation of anti-apoptotic genes, proliferation arrest and melanoma cell death. Furthermore, we show for the first time a chemosensitizing effect of β-catenin inhibition for two different chemotherapeutics in melanoma cell lines. These findings make clear that β-catenin is a promising candidate for novel approaches in the therapy of malignant melanoma.

## Materials and Methods

### Cell Culture

Human metastatic melanoma cell lines were cultured in RPMI 1640 medium with 10% fetal bovine serum (FBS; Biochrom, Berlin), penicillin and streptomycin. The non-metastatic melanoma cell lines were maintained in “2% Tumor” medium composed of MCDB153∶L15 (4∶1) supplemented with 2% FBS and 5 µg/ml insulin, unless otherwise stated. The isolation and culturing of these cell lines have been described elsewhere [51], [52]. These well-defined human melanoma cell lines derived either from human primary melanomas or from melanoma metastases recapitulate *in vitro* and *in vivo* their biological behaviour of the original tumor cells and are therefore representative members of melanoma cells in the different progression stages [53], [54]. The cell lines were kindly gifted by M. Herlyn except for Mewo, SKMel28, SKMel19, MV3 and HCT116 which were purchased from ATCC [55]. Melanocytes, keratinocytes and fibroblasts were isolated from human foreskin after routine circumcision. Melanocytes, fibroblasts and keratinocytes were isolated as described previously [54], [56], [57]. All of the cell lines used in our study were authenticated by sequence analysis of defined genes. The use and culturing of human skin tissues in this study was approved by the medical ethical committee of the University of Tübingen (43/2008B01; 16/2009B02) and was performed in accordance with the Declaration of Helsinki Principles. All patients provided informed written consent.

### Real-time PCR

All cell lines used for expression analyses were cultured 24 hours before RNA extraction in the same medium (RPMI/10%FCS). DNA-free total RNA was extracted with the NucleoSpin RNA II kit (Machery-Nagel, Germany) and 1 µg RNA reverse transcribed with Superscript II using hexamer primers into first strand cDNA. The cDNA was used with the corresponding primers (designed with the help of Primer3 and Universal Probe Library, Roche) into a SYBR green real-time PCR and analyzed in a LightCycler480 (Roche, Mannheim, Germany) by the ΔΔCt-method. The following primer sequences were used:

*Gene* *sense* *antisense*

ACTINB ttgttacaggaagtcccttgcc atgctatcacctcccctgtgtg

FN1 gaataagctgtaccatcgcaaa tgtaaccaccagtctcatgtgg

AXN2 atgattccatgtccatgacg cttcacactgcgatgcattt

### Luciferase Reporter Assay

Melanoma cells (3×10^5^ cells per well) were seeded in 6well plate cavities and grown for 24 h. Cells were transfected using Lipofectamin 2000 (Invitrogen, Heidelberg, Germany) with 2.0 µg Super8xTOPFlash and transferred into 96 well plates. 36 hours after transfection cells luciferase activity was measured in lysates using the Dual Luciferase Reporter Assay (Promega) in a TriStar luminometer (Berthold, Germany). RLU firefly values were background subtracted and normalized to the protein concentration of the lysate.

### Organotypic skin culture

Organotypic skin reconstructs were prepared as described previously [21], [23], [54]. Melanoma cells were either implemented into the fibroblast containing dermal layer (as spheroids) or seeded together with the keratinocytes as an epidermal layer.

### Soft agar growth

Five thousand melanoma cells were seeded into each well of 12well plates together with 0.5ml of 0.5% agar noble (Difco, Heidelberg, Germany) in melanoma cell growth medium. After gelation, 1 ml of growth medium was applied on top and inhibitors were added in the indicated concentrations into the feeding 1 ml. Every treatment regimen was set up in triplicates. After 14 days, the formed colonies were counted in every well and photomicrographs were taken.

### Migration and Invasion Assay

Migration and invasion assays were performed in Boyden chambers coated with or without a Matrigel basement membrane matrix (BD Biocoat Matrigel invasion chambers, BD Biosciences, Heidelberg, Germany) as described previously [23]. Depending on the cell line different cell numbers were subjected into the transwell inserts. After incubation for 20 hours at 37°C the invaded cells were fixed and counted after cell staining with hematoxilin-eosin. The assays were performed in triplicates, six fields were counted per transwell filter and mean cell numbers and standard deviations were calculated.

### Real-time invasion assay

The kinetics of cell invasion was assayed using the xCELLigence Real-Time Cell Analyzer (RTCA DP; Roche). CIM-plate 16 wells were pre-coated with 30 µl of matrigel diluted 1∶10 in DMEM for 1 h at 37°C, then 10,000cells were plated in each well in fibroblast conditioned serum-free DMEM. The lower medium chamber contained fibroblast conditioned DMEM with 10% FCS. Cells were allowed to settle for 30 min at room temperature before being placed in the RTCA DP in a humidified incubator at 37°C with 5% CO_2_. Readings were taken every 15 min for 24 h and plotted curves represent the averages from three independent wells/measurements.

### In vivo melanoma growth assay

For the *in vivo* tumor growth assays, 8×10^5^ 451LU cells stably expressing tetracycline-inducible shRNA against β-catenin were subcutaneously injected into SCID mice. 3 weeks post injection small tumor nodules had developed in all the mice. One group was subsequently fed with 200 µg/ml doxycycline in the drinking water for one week. Tumor size was monitored for six weeks post injection by measuring tumor length, width and height. The tumor volume was calculated using the formula for ellipsoids (V = 4/3π*a*b*c). In case of subcutaneous injection of the radial growth phase melanoma cell line SbCl2, 2×10^6^ cells were injected in 100 ul PBS with 10% matrigel into the flanks of the mice. Subcutaneous tumor nodules were prepared four weeks post injection and the tumor size was determined. All animal experiments were approved by the Regierungspräsidium Tübingen (AZ HT6/06).

### Lentiviral gene transfer

Lentiviral particles were produced in HEK 293T cells (Biocat, Heidelberg, Germany) using the previously described transfer plasmids of Skokowa et al. [58] and the second-generation packaging and envelope plasmids pCMVΔR8.2 and pMD2.G. Melanoma cells were transduced with lentivirus containing supernatants in the presence of 8 µg/ml polybrene and checked for β-catenin knockdown by western blot 4 days after transduction.

### Transmission electron microscopy

Cells were washed, centrifuged and the resulting pellets were fixed for 24 hours in Karnovsky's fixative (3% glutaraldehyde (Sigma), 4% paraformaldehyde (Merck), 0.2 M Na-cacodylate (Merck). After centrifugation, the sediment was embedded in 3% agarose at 37°C and then cooled on ice. Post-fixation was based on 1.0% osmium tetroxide containing 1.5% K-ferrocyanide in Aquadest for 2 hours. After embedding in glycide ether the blocks containing cells were cut using an ultra microtome (Ultracut, Reichert, Vienna, Austria) and ultra-thin sections (30 nm) were examined using a Zeiss Libra120 transmission electron microscope (Carl Zeiss, Oberkochen, Germany) operating at 120 kV.

### Proliferation/Cell viability assay

For the analysis of proliferation and survival of melanoma cells, 2.5×10^3^ cells were seeded into 96-well plates and cultured for the indicated periods of time. After washing, 4-methylumbelliferyl heptanoate in PBS (100 µg/ml) was added and incubated for 1 h at 37°C. Microplates were measured in a fluorescence microplate reader (Berthold, Germany) with Ex355/Em460 nm in quintuplicates. In case of lentiviral shRNA transfer, the increase of DsRed fluorescence which is expressed only in transduced cells was measured from day 2 to day 6 using a microplate fluorescence reader (Berthold, Germany). This fluorescence intensity correlates in a linear way to cell number.

### Apoptosis assays

#### Cell cycle analysis

2×10^5^ cells were permeabilized in ice-cold 70% ethanol for at least four hours and resuspended in 500 µl PBS with 50 U RNAseA (Fermentas, St. Leon-Rot, Germany) and 50 µg/ml of propidium iodide (Sigma, Taufkirchen, Germany). After 30 min cells were analyzed in a LSRII FACS (BD, Heidelberg, Germany).

#### Mitochondrial staining

the following antibodies were used as recommended by the manufacturer: anti-cytochrome c (BD Biosciences, Heidelberg, Germany); anti-COX IV (Abcam, Cambridge, UK).

#### AnnexinV-FITC/PI staining

After the indicated time of culture, 1×10^5^ cells were resuspended in 50 µl binding buffer (0.1 M HEPES, pH 7.4; 1.4 M NaCl; 25 mM CaCl_2_) containing 5 µl of AnnexinV-FITC (BD, Heidelberg, Germany) and stained for 15 min. Then 10 µg/ml of propidium iodide (Sigma, Taufkirchen, Germany) was added for 5 min and the cells were analyzed in a LSRII FACS.

### Caspase 3 activity assay

2×10^4^ cells were seeded into 96 well plates in quintuplicates, treated as indicated and lysed in 50 µl caspase lysis buffer (20 mM HEPES pH 7.4, 84 mM KCl, 10 mM MgCl_2_, 0.2 mM EDTA, 0.2 mM EGTA, 0.5% NP-40). 150 µl reaction buffer containing 50 mM HEPES pH 7.4, 100 mM NaCl, 2 mM CaCl_2_, 5 mM DTT, 10% sucrose, 0.1% CHAPS and 70 µM DEVD-AMC (Enzo Life Sciences, Lörrach) were added to each well and fluorescence was measured every 15 min for one hour in a Tristar microplate reader (Berthold Technologies, Bad Wildbad) at Ex488 nm/Em512 nm.

### Luciferase Reporter Assay

Melanoma cells (8×10^4^ cells per well) were seeded in 24well plates and grown for 24 h. Cells were transfected using Lipofectamin 2000 (Invitrogen, Heidelberg, Germany) with 0.4 µg Super8xTOPFlash. 36 hours after transfection cells luciferase activity was measured using the Dual Luciferase Reporter Assay (Promega) in a TriStar luminometer (Berthold, Germany). RLU firefly values were background subtracted and normalised to protein content.

### Western Blot analysis

30 µg of lysate were subjected to SDS PAGE and blotted onto PVDF membrane (Roche, Mannheim, Germany). The following antibodies were used: anti-Mcl-1, anti-Bcl-xl (both from BD Biosciences, Heidelberg, Germany), anti-Bcl-2, anti- β-catenin, anti-Cyclin D1, anti-C-myc (Cell Signaling, Frankfurt, Germany), anti- β-Actin, anti-CK1α and anti-LaminB (Santa Cruz, Heidelberg, Germany). For preparation of nuclear protein extracts the cells were trypsinised and lysed in a hypotone buffer containing 0.5% NP-40. The detergent insoluble fraction containing nuclei was further treated with a hypertone buffer. Semiquantification of western blots was done with the freeware software tool scion image (NIH image, http://rsb.info.nih.gov/nih-image/) by using the actin signal as a reference.

### Statistical analysis

Statistical analyses were performed with a two-tailed unpaired t-test. P-values<0.05 were considered statistically significant. Response surface analysis of melanoma cells treated with combinations of pathway inhibitors and chemotherapeutic agents was performed as previously described [26]. Briefly, the effects of drug combinations (z axis) were plotted against the concentrations of the single drugs (x-, y-axes). The surface representing additive effects was developed with the dose-response data, curve fitted to the hyperbolic equation: effect = (maximal effect×concentration)/(maximal effect+concentration of half-maximal effect).

## Supporting Information

Figure S1 **(A) Pharmacological β-catenin inhibition reduces β-catenin signaling and protein level in melanoma cells.** SkMel28 melanoma cells were treated with 1 µM of the inhibitor PKF115–584 and nuclear β-catenin expression was analyzed by confocal immunofluorescence microscopy over time (nuclei: YOPRO-1 green; β -catenin: red) (left panel). In addition, β-catenin-TCF/LEF signaling was determined by a Super8xTOPflash reporter assay. PKF115–584 inhibits the signaling in a concentration dependent manner (right panel). Asteriks denote significant differences as measured by t-test with one asteriks being p<0.05, two asteriks being p<0.01 and three asterisk being p<0.005 (t-test). **(B) PKF115–584 treatment reduces protein levels of β-catenin, Cyclin D1, Mcl-1 and Bcl-xl.** Shown are western blot analyses of melanocytes (NHM), WM 35, WM115, WM793, 451LU, 1205LU, Mewo and SKMel28 melanoma cells treated for 24 hours with increasing concentrations of PKF115–584 (0–2 µM). Lysates were analyzed for expression of β-catenin, cyclin D1, Mcl-1, Bcl-xl and actin. The colorectal carcinoma cell line HCT116 served as a reference cell line.
(TIF)

Figure S2 **(A) Inhibition of β-catenin expression by shRNA reduces β-catenin protein level in melanoma cells.** SkMel28 and 451LU melanoma cells were lentivirally transduced with shRNA against β-catenin or luciferase as a control. Total protein lysates were produced at the indicated time points and 30 µg of lysate were subjected to a β-catenin western blot (two top panels). On the right semiquantification of the western blots (β-catenin protein expression normalized to actin) is shown. **(B) Inhibition of β-catenin expression by inducible shRNA reduces nuclear β-catenin protein level.** Two clones stably expressing a Docycycline-inducible shRNA against β-catenin were generated from the parental cell lines SKMel28 and 451LU with pcDNA6/TR and pTER-β-catenin. Both cell lines were induced with 1 µg/ml doxycycline for the indicated time points. Protein isolates of the induced clones revealed a β-catenin downregulation in nuclear protein fractions after 4 days (shown is the 451LU clone). As shown in the right diagram β-catenin RNA expression measured by real-time PCR is reduced after doxycycline treatment in the clones expressing shRNA against β-catenin.
(TIF)

Figure S3 **PKF115–584 treatment induces apoptosis in metastatic melanoma cells.** (**A**) *AnnexinV-FITC/PI staining:* SKMel28 melanoma cells were treated for 24–72 hours with increasing concentrations of PKF115–584 (0–2 µM) and stained with AnnexinV plus PI to detect apoptotic and necrotic cells in a flow cytometry analysis. In a time and concentration dependent manner the melanoma cells got AnnexinV positive (apoptotic) before ending as AnnexinV and PI double positive dead cells. Only a small amount of cells were necrotic as indicated by the early appearance of AnnexinV/PI +/+ cells. (**B**) *Caspase 3 activity:* 451LU and SKMel28 melanoma cells were treated with 1 µM PKF115–584 for 24 h or 48 h and cell lysates were used to measure the DEVD-AMC cleavage activity as indicator for activated caspase 3. The treatment induced a significant (p<0.01) higher caspase activity of 10–30-fold, normalized to untreated controls. A staurosporine treatment for 4 h served as a positive control.
(TIF)

Figure S4 **shRNA against β-catenin reduces melanoma cell growth of metastatic melanoma cell lines (raw data to** **Figure 1B** **).** Different melanoma cell lines were lentivirally transduced with shRNA against β-catenin or a non-silencing control (both with RFP as a marker). The proliferation of the metastatic melanoma cell lines 451LU, SKMel28, 1205LU and Mewo ceased. Shown are the measured fluorescence values of RFP expression of transduced cells over time, normalized to the first day of measurement (day 2 after transduction). Increased RFP values are indicative for an increase in cell number/cell proliferation.
(TIF)

Figure S5 **Ablation of β-catenin activity in melanoma cells inhibits migration, invasion (raw data to** **Figure 2A** **).** Melanocytes (NHM) and non-metastatic melanoma cell lines or metastatic melanoma cell lines were used for Boyden-chamber experiments. Matrigel coated transwells were used to perform invasion assays and non-coated transwells to assay the cell migration capability after treatment with 0.5 µM PKF115–584 for 12 hours or after the induced expression of shRNA against β-catenin for 24 hours. Cell viability was not affected as seen by trypan blue staining. Shown is the number of counted cells per field. All reductions in migration and invasion were significant except for NHM (p<0.05).
(TIF)
