# Supplementary figures and images for: β-Catenin Signaling Increases during Melanoma Progression and Promotes Tumor Cell Survival and Chemoresistance
Source: PLoS One. 2011 Aug 17;6(8):e23429. doi: 10.1371/journal.pone.0023429 (PMC3157382; doi:10.1371/journal.pone.0023429)

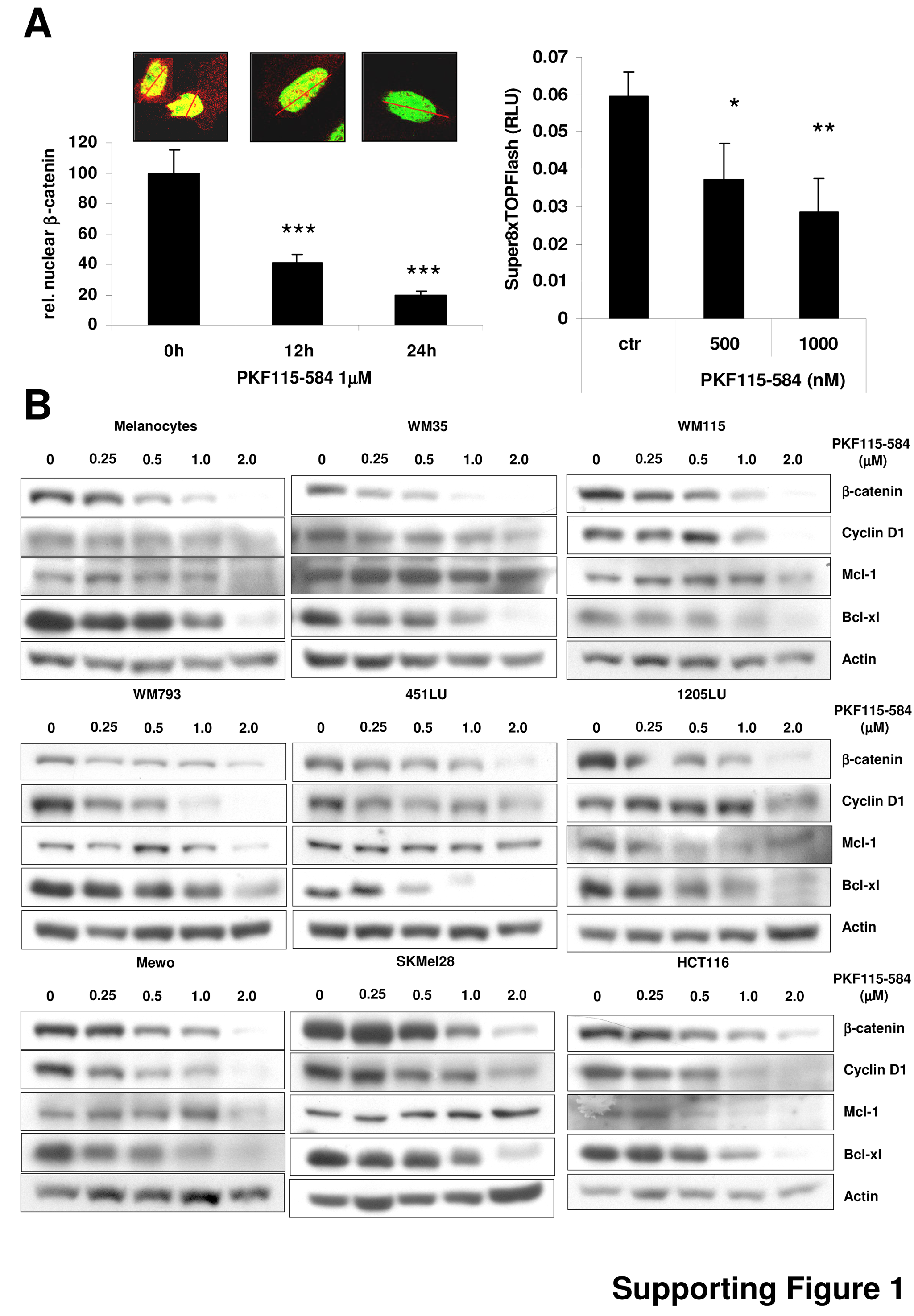

Supplement: Figure S1 — (A) Pharmacological β-catenin inhibition reduces β-catenin signaling and protein level in melanoma cells. SkMel28 melanoma cells were treated with 1 µM of the inhibitor PKF115–584 and nuclear β-catenin expression was analyzed by confocal immunofluorescence microscopy over time (nuclei: YOPRO-1 green; β -catenin: red) (left panel). In addition, β-catenin-TCF/LEF signaling was determined by a Super8xTOPflash reporter assay. PKF115–584 inhibits the signaling in a concentration dependent manner (right panel). Asteriks denote significant differences as measured by t-test with one asteriks being p<0.05, two asteriks being p<0.01 and three asterisk being p<0.005 (t-test). (B) PKF115–584 treatment reduces protein levels of β-catenin, Cyclin D1, Mcl-1 and Bcl-xl. Shown are western blot analyses of melanocytes (NHM), WM 35, WM115, WM793, 451LU, 1205LU, Mewo and SKMel28 melanoma cells treated for 24 hours with increasing concentrations of PKF115–584 (0–2 µM). Lysates were analyzed for expression of β-catenin, cyclin D1, Mcl-1, Bcl-xl and actin. The colorectal carcinoma cell line HCT116 served as a reference cell line. (TIF) [file pone.0023429.s001.tif]

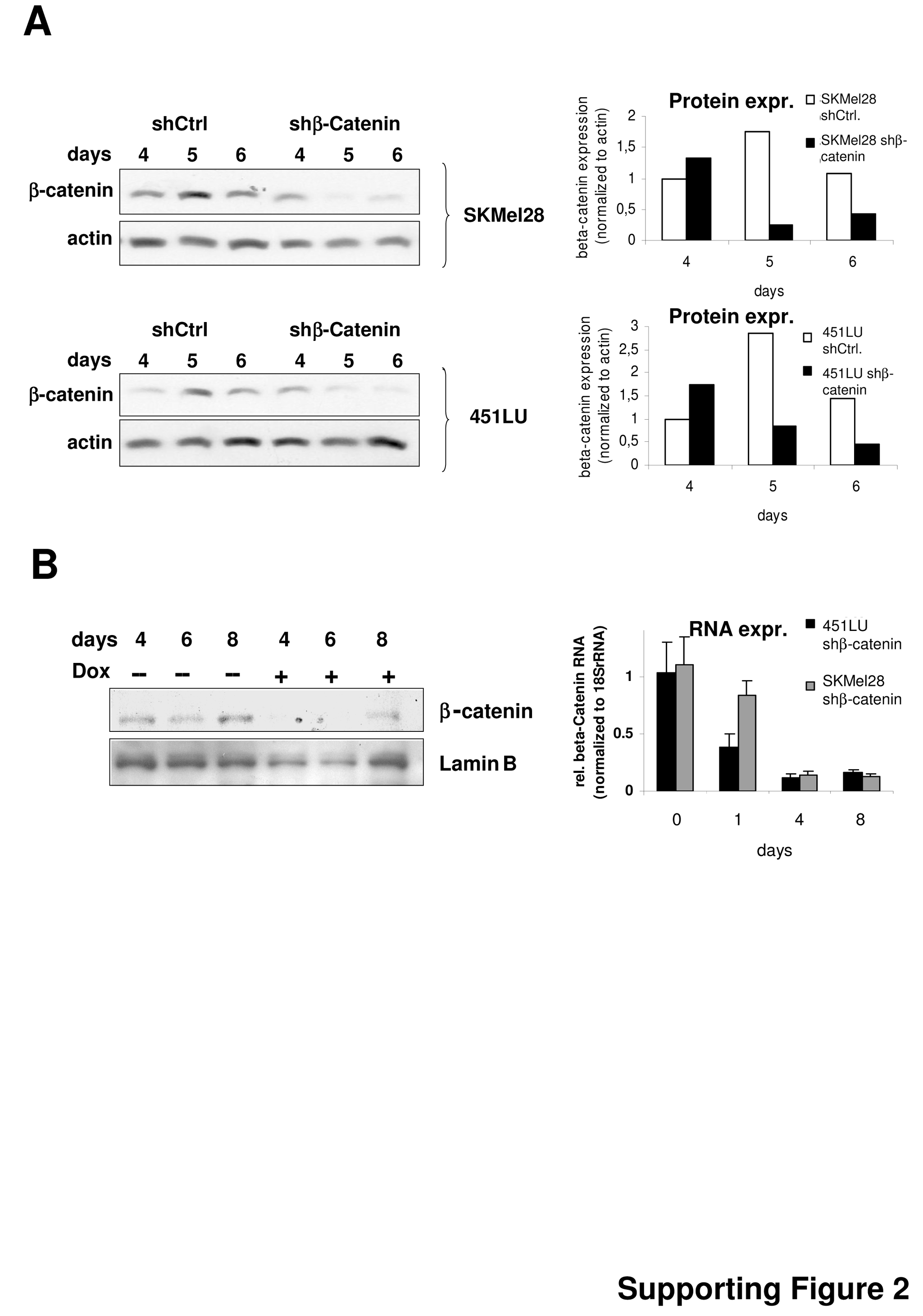

Supplement: Figure S2 — (A) Inhibition of β-catenin expression by shRNA reduces β-catenin protein level in melanoma cells. SkMel28 and 451LU melanoma cells were lentivirally transduced with shRNA against β-catenin or luciferase as a control. Total protein lysates were produced at the indicated time points and 30 µg of lysate were subjected to a β-catenin western blot (two top panels). On the right semiquantification of the western blots (β-catenin protein expression normalized to actin) is shown. (B) Inhibition of β-catenin expression by inducible shRNA reduces nuclear β-catenin protein level. Two clones stably expressing a Docycycline-inducible shRNA against β-catenin were generated from the parental cell lines SKMel28 and 451LU with pcDNA6/TR and pTER-β-catenin. Both cell lines were induced with 1 µg/ml doxycycline for the indicated time points. Protein isolates of the induced clones revealed a β-catenin downregulation in nuclear protein fractions after 4 days (shown is the 451LU clone). As shown in the right diagram β-catenin RNA expression measured by real-time PCR is reduced after doxycycline treatment in the clones expressing shRNA against β-catenin. (TIF) [file pone.0023429.s002.tif]

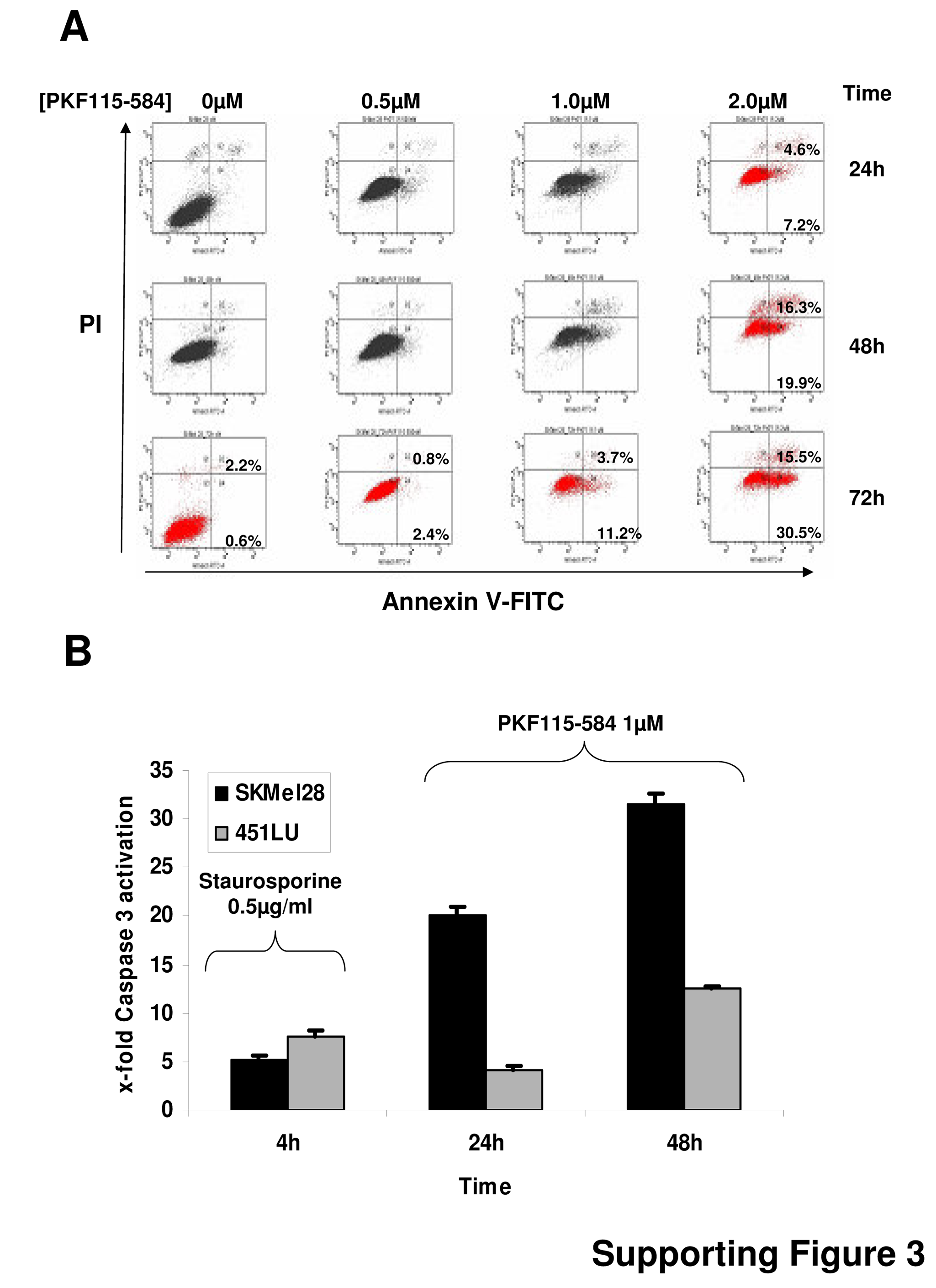

Supplement: Figure S3 — PKF115–584 treatment induces apoptosis in metastatic melanoma cells. (A) AnnexinV-FITC/PI staining: SKMel28 melanoma cells were treated for 24–72 hours with increasing concentrations of PKF115–584 (0–2 µM) and stained with AnnexinV plus PI to detect apoptotic and necrotic cells in a flow cytometry analysis. In a time and concentration dependent manner the melanoma cells got AnnexinV positive (apoptotic) before ending as AnnexinV and PI double positive dead cells. Only a small amount of cells were necrotic as indicated by the early appearance of AnnexinV/PI +/+ cells. (B) Caspase 3 activity: 451LU and SKMel28 melanoma cells were treated with 1 µM PKF115–584 for 24 h or 48 h and cell lysates were used to measure the DEVD-AMC cleavage activity as indicator for activated caspase 3. The treatment induced a significant (p<0.01) higher caspase activity of 10–30-fold, normalized to untreated controls. A staurosporine treatment for 4 h served as a positive control. (TIF) [file pone.0023429.s003.tif]

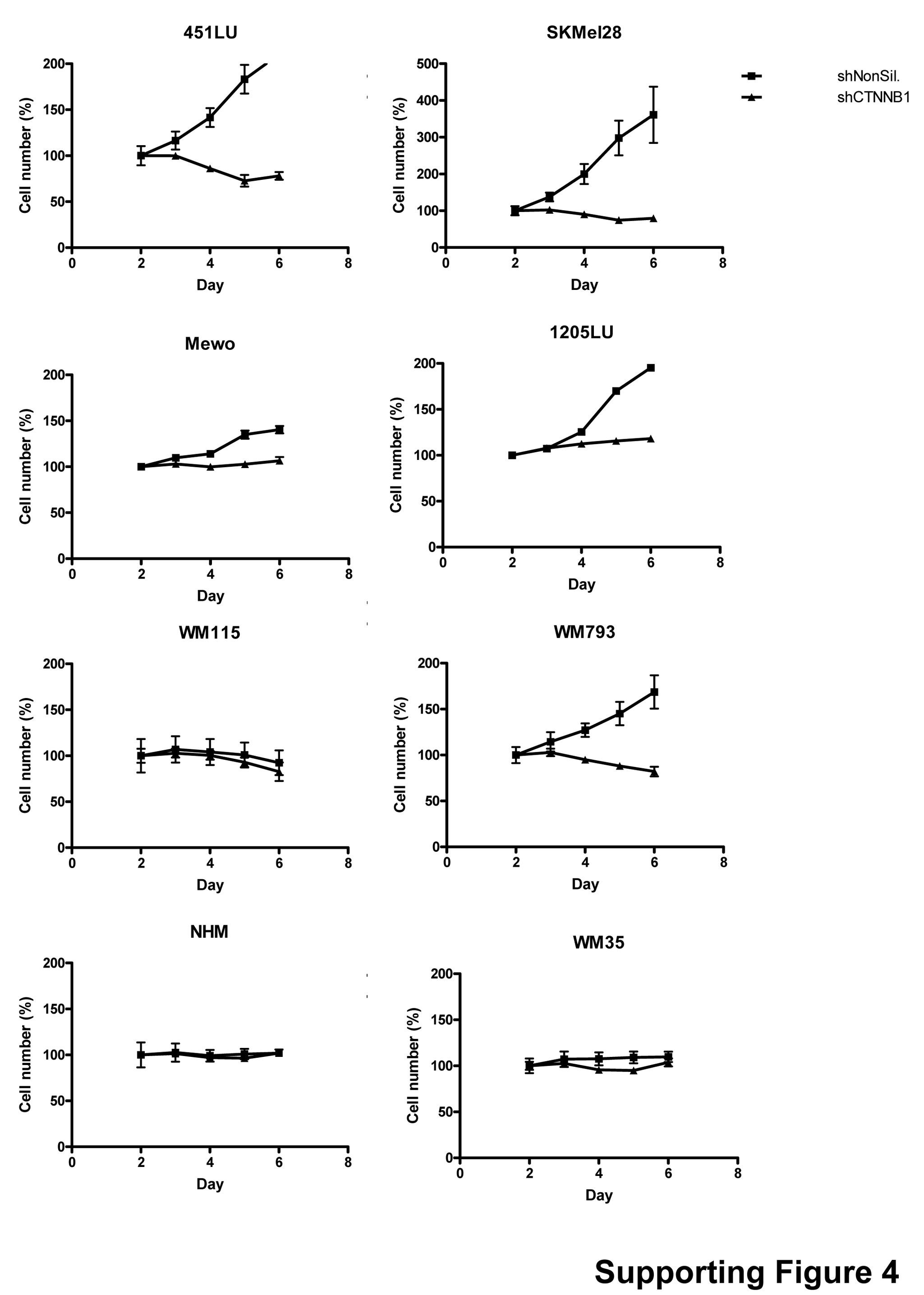

Supplement: Figure S4 — shRNA against β-catenin reduces melanoma cell growth of metastatic melanoma cell lines (raw data to Figure 1B ). Different melanoma cell lines were lentivirally transduced with shRNA against β-catenin or a non-silencing control (both with RFP as a marker). The proliferation of the metastatic melanoma cell lines 451LU, SKMel28, 1205LU and Mewo ceased. Shown are the measured fluorescence values of RFP expression of transduced cells over time, normalized to the first day of measurement (day 2 after transduction). Increased RFP values are indicative for an increase in cell number/cell proliferation. (TIF) [file pone.0023429.s004.tif]

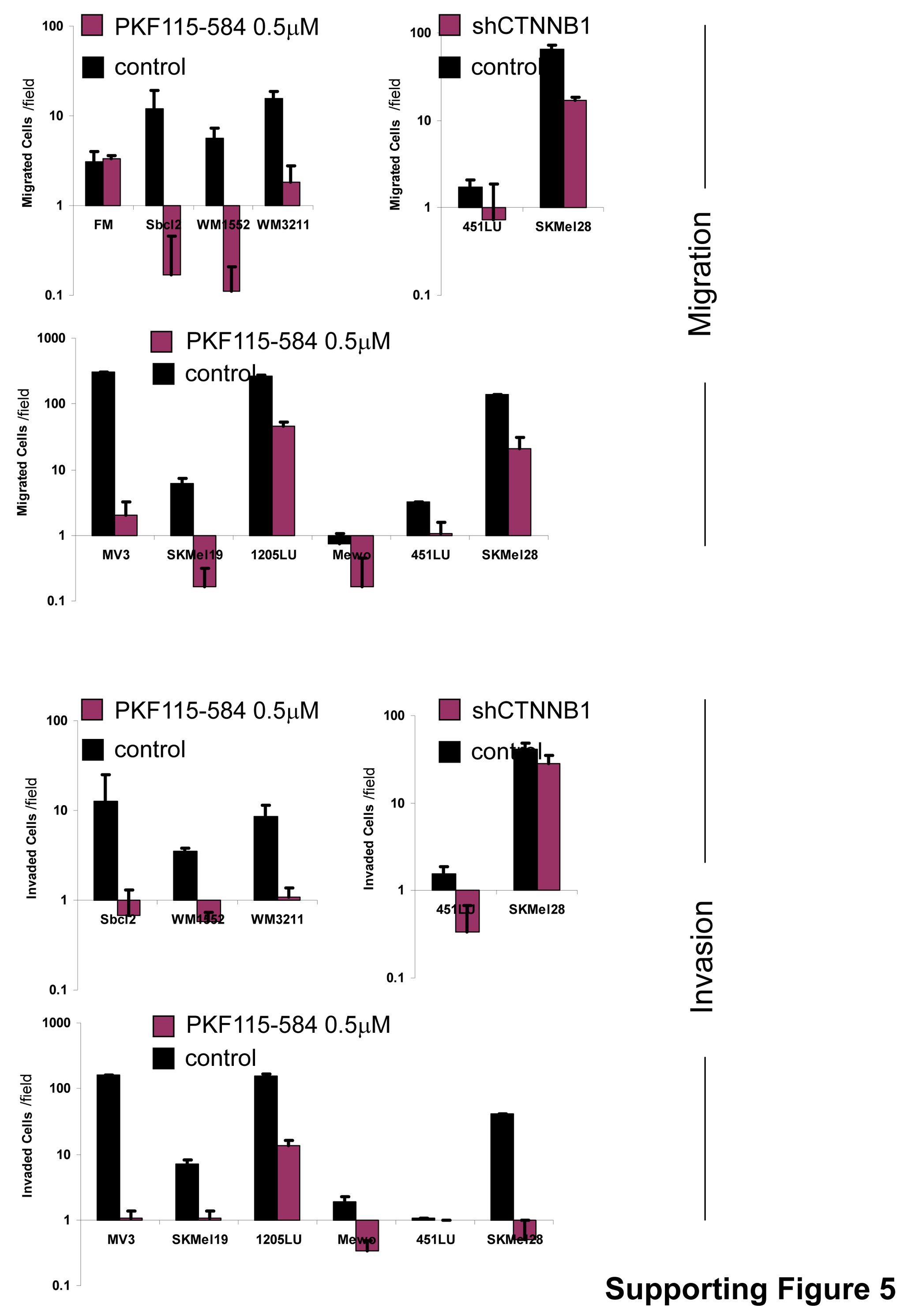

Supplement: Figure S5 — Ablation of β-catenin activity in melanoma cells inhibits migration, invasion (raw data to Figure 2A ). Melanocytes (NHM) and non-metastatic melanoma cell lines or metastatic melanoma cell lines were used for Boyden-chamber experiments. Matrigel coated transwells were used to perform invasion assays and non-coated transwells to assay the cell migration capability after treatment with 0.5 µM PKF115–584 for 12 hours or after the induced expression of shRNA against β-catenin for 24 hours. Cell viability was not affected as seen by trypan blue staining. Shown is the number of counted cells per field. All reductions in migration and invasion were significant except for NHM (p<0.05). (TIF) [file pone.0023429.s005.tif]
